# PEEK surface modification by fast ambient-temperature sulfonation for bone implant applications

**DOI:** 10.1098/rsif.2018.0955

**Published:** 2019-03-06

**Authors:** Weigeng Wang, C. J. Luo, Jie Huang, Mohan Edirisinghe

**Affiliations:** Department of Mechanical Engineering, University College London, Torrington Place, London WC1E 7JE, UK

**Keywords:** polyetheretherketone, PEEK, sulfonation, bone implant, ambient-temperature surface modification

## Abstract

We develop a simple, fast and economical surface treatment under ambient temperature to improve the hydrophilicity and osteoconductivity of polyetheretherketone (PEEK) for bone implant applications. A major challenge in bone implants is the drastic difference in stiffness between traditional implant materials (such as titanium and stainless steel) and human bone. PEEK is biocompatible with an elastic modulus closely matching that of human bone, making it a highly attractive alternative. However, its bio-inert and poorly hydrophilic surface presents a serious challenge for osseointegration. Sulfonation can improve hydrophilicity and introduce bioactive sulfonate groups, but PEEK sulfonation has traditionally been applied for fuel cells, employing elevated temperatures and long reaction times to re-cast PEEK into sulfonated films. Little research has systematically studied PEEK surface modification by short reaction time (seconds) and ambient-temperature sulfonation for biomedical applications. Here, we investigate three ambient-temperature sulfonation treatments under varying reaction times (5–90 s) and evaluate the hydrophilicity and morphology of 15 modified PEEK surfaces. We establish an optimal treatment using 30 s H_2_SO_4_ followed by 20 s rinsing, and then 20 s immersion in NaOH followed by 20 s rinsing. This 30 s ambient-temperature sulfonation is found to be more effective than conventional plasma treatments and reduced PEEK water contact angle from 78° to 37°.

## 1. Introduction

Bone grafts and implants have important clinical significance, especially for an ageing population. The human bone is capable of complete regeneration if a suitable support such as an osteoconductive scaffold is provided for new osteoblast growth. One of the greatest challenges faced with bone implants is the mechanical mismatch between the implant materials available and the regenerating bone. For example, Young's modulus of human trabecular and cortical bone is between 10.4 ± 3.5 and 20.7 ± 1.9 GPa [1,2], whereas Young's moduli of materials widely used for bone implant applications such as titanium, stainless steel and cobalt chromium alloys are approximately 110 GPa, 180 GPa and 210 GPa, respectively [3,4]. When an implant is significantly stiffer than the host bone, the latter bears a lesser load than the implant. The lack of load stimulation over time causes the bone to weaken and become less dense, a process known as stress shielding [5,6]. Further exacerbating the problem, the softer host tissue also develops a fibrous encapsulation at the interface between the bone and the harder implant. The fibrous tissue reduces osseointegration and improperly shifts the implant, creating local abrasion of the bone tissue and the implant material. This process, also known as fretting, can cause implant failure [7].

These clinical issues associated with traditional bone implants have led to a growing interest in alternatives such as shape memory nitinol (NiTi) metal alloys [8] and polymeric materials which can better match the stiffness of human bone, thereby reducing stress shielding and fretting effects. One of the most promising alternatives for dental, spinal and large bone trauma applications is polyetheretherketone (PEEK) [9]. PEEK is a semi-crystalline, polycyclic thermoplastic. In its unmodified state, its Young's modulus is around 3.6 GPa; when reinforced with carbon fibres, its Young's modulus improves to around 18 GPa, close to that of human cortical bone [9,10]. Moreover, PEEK is biocompatible, chemically and physically stable, and features many further advantages when compared with traditional metal and ceramic implants. These include radiolucency, high strength to weight ratio, ability to provide physiologically relevant colours (from white to tooth coloured to gingiva coloured), low cost and ready machinability for patient-specific designs [9,11]. Furthermore, PEEK is currently used as a clinical implant with proven non-cytotoxicity and capability of bone induction [12,13].

However, PEEK is bio-inert and relatively hydrophobic, giving rise to poor osseointegration that hampers its long-term clinical success [14]. Osseointegration between the implant surface and bone tissue is a principal indicator for a successful orthopaedic implant [15]. To achieve an osseointegrated implant, the surface of the implant material should enable effective adhesion of osteoblasts—cells that assist in building mineralized bone [14]. Studies have shown that the adhesion, proliferation and differentiation of osteoblasts are strongly influenced by hydrophilicity, roughness, porosity and the presence of bioactive groups on the implant surface [16]. Consequently, surface modification is a major procedure to activate the surface of PEEK implants for osteoblast adhesion.

In particular, sulfonation can be used to increase the hydrophilicity of PEEK by introducing charged sulfonate (−SO_3_−) groups into the polymer backbone [17]. Furthermore, polymeric scaffolds bearing sulfonate groups, such as polysulfonate copolymer hydrogels, have been found to increase non-specific interactions between the scaffold and the glycocalyx molecules found on the cell outer membrane, thereby enhancing the adhesion and proliferation of osteoblast-like cells [18–20].

Sulfonation of PEEK can be applied before or after polymerization. Sulfonating the PEEK monomers prior to polymerization can achieve a high degree of sulfonation, but the resultant material has poor mechanical stability [21]. Hence, post-polymerization sulfonation is preferable in bone implant applications, where the mechanical integrity is critically important. Research on post-polymerization sulfonation of PEEK has thus far focused on the complete re-casting of PEEK into sulfonated PEEK membranes for fuel cell applications. Sulfonation has also traditionally relied on long reaction times (greater than 30 min) under elevated temperatures (greater than 25°C) to achieve suitable hydrophilicity in the sulfonated membrane [17,22,23]. Recently, surface-modified sulfonated PEEK (SPEEK) by a brief duration sulfonation method (5 min under supersonic stirring) has been shown to be biocompatible and successfully induced pre-osteoblast functions including cell adhesion, proliferation and osteogenic differentiation *in vitro* as well as substantially enhanced osseointegration and bone–implant bonding strength *in vivo* [24]. Nonetheless, little previous literature has systematically examined short reaction time post-polymerization sulfonation methods to modify the surface of PEEK for bone implant applications [25].

The aim of this work is, therefore, to systematically develop fast ambient-temperature sulfonation that can effectively improve the hydrophilicity of the surface of PEEK and introduce bioactive nano-topography for bone implant applications. We test three ambient-temperature (22°C) sulfonation treatments and study an extensive array of modified PEEK surfaces. Five different reaction times (5–90 s) were tested for each of the three treatments and an optimal combination of variables was established to activate PEEK surfaces for future large-scale bone implant applications.

## 2. Material and methods

### 2.1. Materials

Concentrated sulfuric acid (H_2_SO_4_, 95–98%, molecular weight 98.079 g mol^−1^), ethanol (99%) and sodium hydroxide (NaOH) were obtained from Sigma–Aldrich (Poole, UK). A saturated aqueous solution of 6 wt% NaOH was prepared under ambient conditions of 20°C and 1 atmospheric pressure. All chemicals were of analytical grade and used as received. Industrial grade unfilled virgin PEEK sheets with a thickness of 2.0 mm were supplied by Goodfellow Cambridge Ltd, UK. The PEEK sheet was cut into 2 × 1 cm pieces.

### 2.2. Sulfonation treatments of PEEK

**Figure 1.**
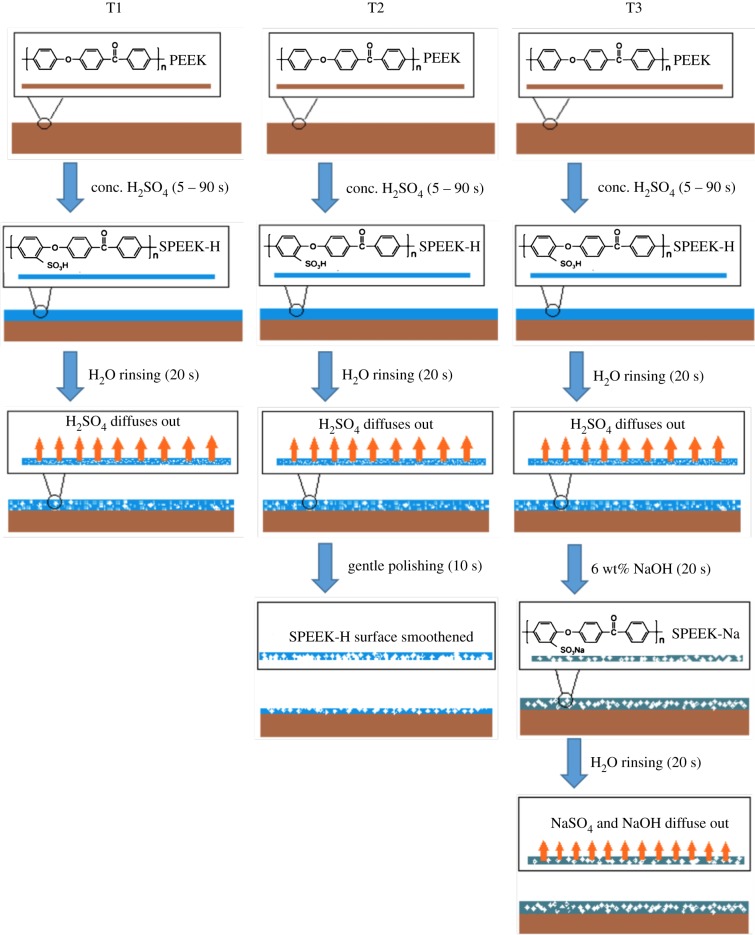
A schematic diagram showing treatment methods. T1: treatment 1, T2: treatment 2 and T3: treatment 3. (Online version in colour.)

Three kinds of sulfonation treatments were tested (figure 1). Treatment 1: water immersion and rinsing after sulfonation; the virgin PEEK samples were immersed in high concentration (95–98%) sulfuric acid for a controlled duration followed by immersion in distilled water for 20 s to remove the residual sulfuric acid on the surface (figure 1, T1). Treatment 2: 20 s water immersion and rinsing after sulfonation followed by hand polishing with soft laboratory tissue for 10 s (figure 1, T2). Treatment 3: 20 s water immersion and rinsing after sulfonation, followed by 20 s immersion in 6 wt% NaOH, followed by 20 s washing in distilled water (figure 1, T3).

The sulfonation reaction time was studied for all treatment methods by varying the duration of immersion in sulfuric acid between 5 s, 10 s, 30 s, 60 s and 90 s. The final pH of all treatments was measured using a calibrated pH meter (ELIT ion analysers; NICO 2000 Ltd, UK) to determine the presence of residual reactants. All samples were dried for 3 days under ambient temperature and 1 atmospheric pressure prior to analyses.

### 2.3. Compositional characterization

Fourier transform infrared spectroscopy (FTIR spectrometer; Spectrum Two; PerkinElmer, Waltham, MA, USA) was used to confirm the presence of sulfonated groups after the treatments by analysing the functional groups of the polymer in the treated PEEK samples and the untreated control samples. The measurements were interpreted using NIOS2 Main software. Each sample was scanned 20 times at a resolution of 4 cm^–1^ over a range of 400–4000 cm^−1^.

### 2.4. Morphological characterization

The surface morphology was evaluated using scanning electron microscopy (SEM; Hitachi S-3400 N; Hitachi High-Technologies Scientific Instruments, Wokingham UK), at an accelerating voltage of 5 kV. Prior to observation, each sample was sputtered with gold in a Quorum Q150R ion sputter (Quorum Technologies, Lewes, UK) for 90 s. Porosity was analysed and averaged based on 30 measurements from the SEM images using Image Tool (UTHSCSA; Image Tool Version 2; University of Texas, TX, USA).

### 2.5. Wettability and water contact angle characterization

The surface properties were analysed using contact angle measurements. The contact angle is defined as the angle between the solid surface and the liquid–vapour interface when a liquid droplet is deposited on a solid surface (electronic supplementary material, figure S1). Here, we use the contact angle between water and PEEK to characterize the wettability of the PEEK samples.

PEEK surface wettability was measured using deionized water at 22°C based on contact angle measurements taken using the static sessile drop method. A droplet of 67 ± 3 µl of deionized water suspended from a needle (Kruss needle specification—model NE62, OD = 1 mm, ID = 0.82 mm) was allowed to fall freely onto the substrate surface. A high-speed camera recorded the freefall motion. The angle made between the surface and the water droplet was analysed using a DSA10 instrument fitted with a high-speed camera (electronic supplementary material, figure S1b; DSA10-Mk2 drop shape analysis contact angle system; Krüss, Germany). Three measurements were taken from the surface of each sample, and the droplet angle was measured using a circular algorithm technique. The water contact angle on an untreated virgin PEEK surface was used as the control.

#### 2.5.1. Corrected water contact angle based on Wenzel equation

To take into account the porous roughened surface of the treated samples, the water contact angles of treated samples were corrected using the Wenzel equation, cosθ* *=* *R* cos*θ*, where *θ* and θ* are, respectively, the measured contact angle and the corrected contact angle on the rough surface (electronic supplementary material, figure S2a,b) and *R* is the ratio of the roughened wet surface area (*A*_t_) to its projection on the apparent solid plane (*A*_s_) (*R* = *A*_t_/*A*_s_) [26]. *A*_s_ is the flat sample area. *A*_t_ is the roughened wet surface area and *A*_t_ = *nA*_p_ + *A*_f_, where *n* is the number of pores on the surface and *A*_p_ is the open pore surface area, which is simplified to hemispheres; hence, *A*_p_ = 2*πr*_p_^2^, where *r*_p_ is the average pore diameter measured based on SEM images. *A*_f_ is the area surrounding the pores on the PEEK surface (electronic supplementary material, figure S2c), and *A*_f_ = *A*_s_ − *A*_p_′, where *A*_p_′ is the total pore size on the projected plane; hence, *A*_p_′ = *n*π*r*_p_^2^. Only the first layer of pores was considered for the Wenzel wetting scenario; *n* and *r*_p_ are averaged based on at least 30 measurements.

### 2.6. Mechanical testing

Treated samples and untreated PEEK (control) were tested by a three-point bending test carried out using a Hounsfield H1KS Benchtop Tester (UK). Samples were tested in sextets and results were read from QMat materials testing and analysis software (Tinius Olsen, UK). The tensile stress–strain curves and Young's moduli of various samples were calculated and plotted following the literature [27].

## 3. Results and discussion

Surface chemistry, nano-topography, porosity and roughness are key features that influence optimal osteoconduction [28]. Three ambient-temperature sulfonation treatments (figure 1) were designed to study their effectiveness in improving the hydrophilicity and surface morphology of PEEK for bone implant applications.

**Figure 2.**
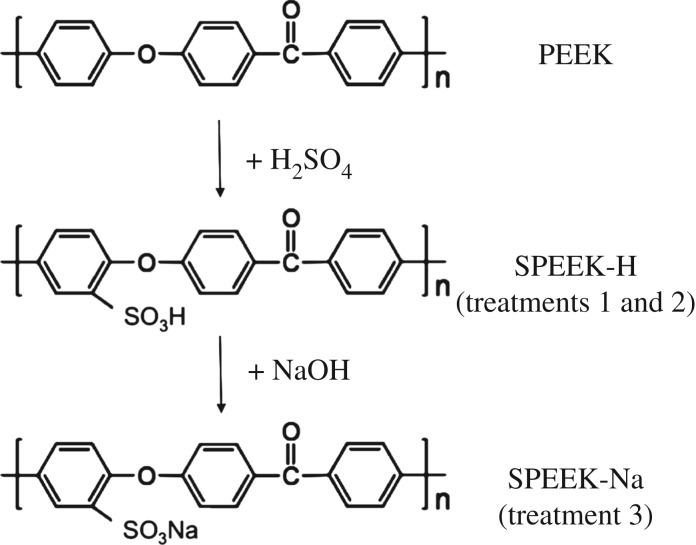
Sulfonation of PEEK (PEEK to SPEEK in treatments 1 and 2) and neutralization of the sulfonated PEEK (SPEEK-H to SPEEK-Na in treatment 3) under ambient conditions.

PEEK sulfonation is a second-order electrophilic reaction; under the mild ambient conditions used in this work, the hydroquinone ring unit beside the ether bridge would be preferentially sulfonated [29]. Treatments 1 and 2 introduced charged sulfonate groups to the PEEK aromatic ring, converting PEEK to sulfonated PEEK (SPEEK–H); treatment 3 further converted SPEEK–H to its sodium salt form, SPEEK–Na (figure 2).

### 3.1. Fourier transform infrared spectroscopy analysis

**Figure 3.**
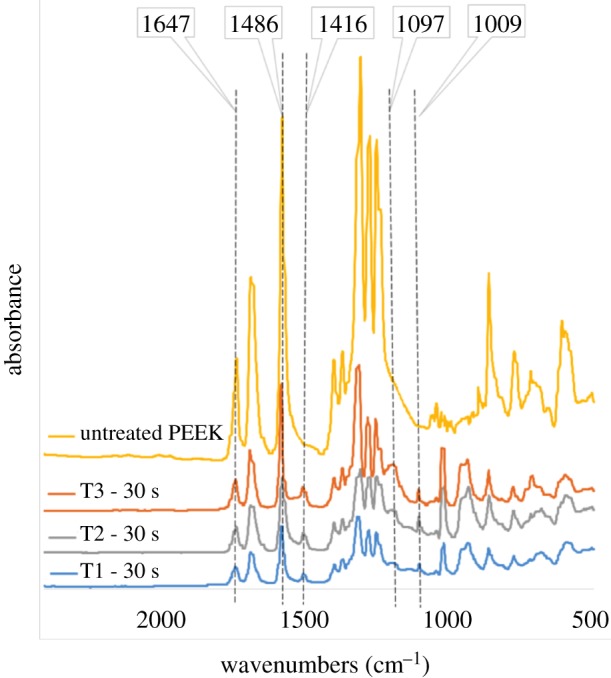
FTIR spectra of untreated PEEK and treated samples from T1, T2 and T3 under the same sulfonation reaction time of 30 s. (Online version in colour.)

The presence of sulfonate groups on the treated samples was confirmed using FTIR. Figure 3 shows a comparison of the FTIR spectra of virgin untreated PEEK and treated samples from T1, T2 and T3 at the same reaction time of 30 s. The backbone carbonyl band at 1647 cm^−1^ appeared unchanged between the virgin PEEK and the treated samples. In all samples from T1 to T3, a new peak appeared at 1416 cm^−1^ in comparison with the untreated PEEK, owing to new sulfonate substitution at the aromatic C–C band at 1486 cm^−1^. Moreover, two new absorption peaks in the treated samples appeared at 1009 cm^−1^ and 1097 cm^−1^, and were, respectively, assigned to the O=S=O symmetric and asymmetric vibrations [30,31].

**Figure 4.**
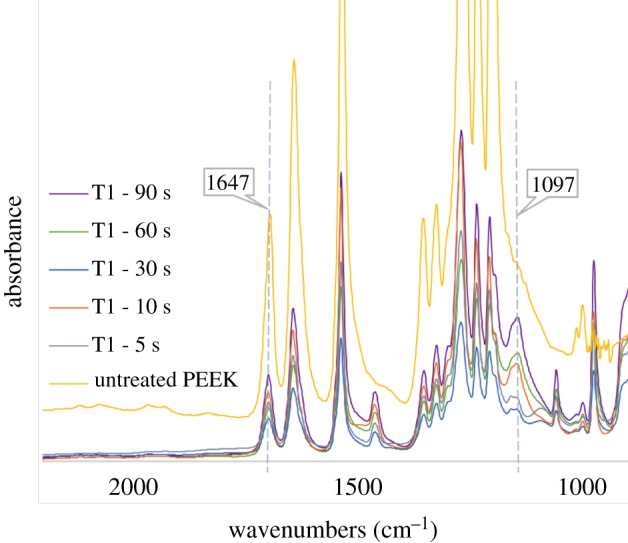
FTIR spectra of untreated PEEK and sulfonated T1 samples under increasing sulfonation reaction times of 5 s, 10 s, 30 s, 60 s and 90 s. (Online version in colour.)

Sulfonation reaction time strongly influences the modification process. Longer reaction time leads to a higher degree of sulfonation and introduces more polar sulfonate groups [17]. The sulfonation reaction time for each treatment was varied between 5 and 90 s. The FTIR results confirmed that the increasing sulfonation reaction time has resulted in increased sulfonate substitution (figure 4). In particular, the O = S = O band at 1097 cm^−1^ that appeared upon sulfonation was observed to intensify with increasing reaction time from 5 s to 90 s (figure 4), while no change was observed in the backbone carbonyl peak at 1647 cm^−1^ [30].

### 3.2. Effect of sulfonation on surface morphology

**Figure 5.**
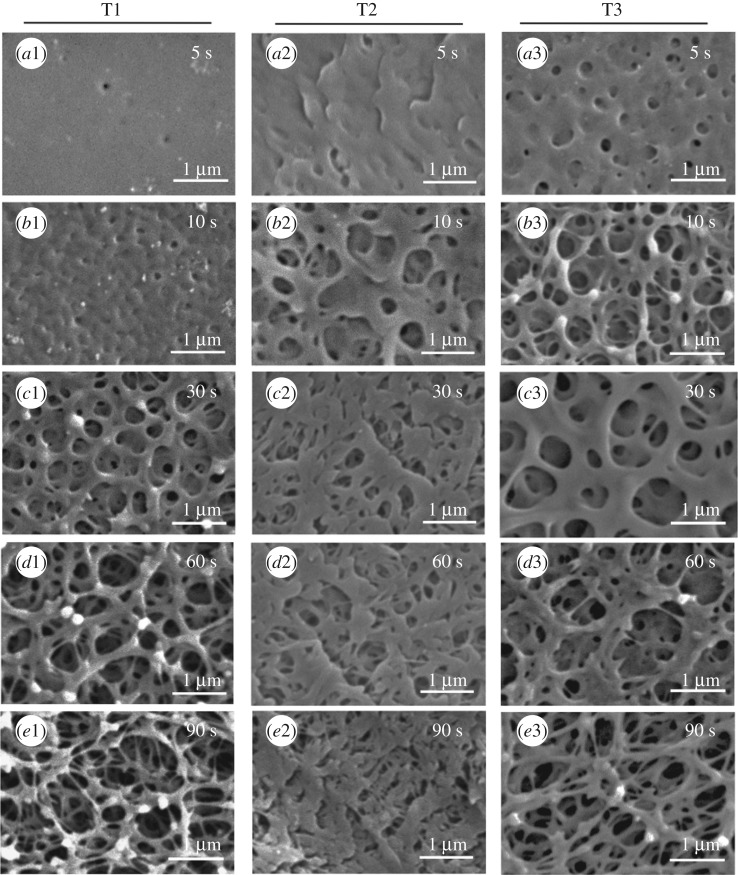
SEM images of PEEK surfaces after each treatment. Left panel: treatment 1. Middle panel: treatment 2. Right panel: treatment 3. (*a*1–3): 5 s, (*b*1–3): 10 s, (*c*1–3): 30 s, (*d*1–3): 60 s, (*e*1–3): 90 s. Scale bars: 1 µm. Magnifications: 27 000×.

**Figure 6.**
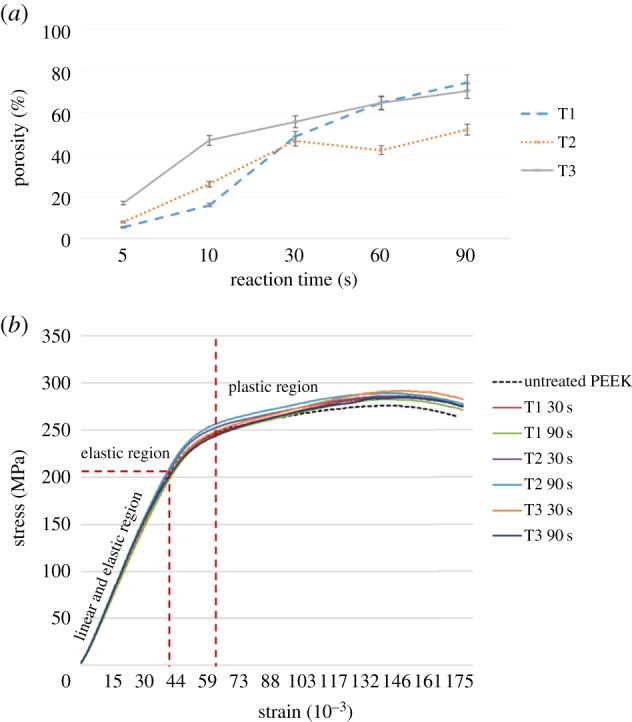
(*a*) Porosity of PEEK samples with increasing sulfonation reaction time. T1: treatment 1. T2: treatment 2. T3: treatment 3. (*b*) Stress–strain curves of treated versus untreated PEEK samples, which confirm that the treatments do not affect the mechanical properties of PEEK samples. (Online version in colour.)

Scanning electron micrographs revealed significant change on the surface morphology of PEEK samples after sulfonation (figure 5). In particular, the surface was found to become increasingly porous with increasing reaction time for all treatments (figure 6). The highest porosity was found at 74.5 ± 1.0% from treatment 1 with the longest reaction time at 90 s (figure 6). This is because, when exposed to sulfuric acid, PEEK dissolution and sulfonation occur concurrently [32]. The main interaction between the molecular chains in amorphous sulfonated PEEK for both SPEEK–H (treatments 1 and 2) and SPEEK–Na (treatment 3) is the electrostatic forces between charged sulfonate groups, which can also strongly interact with water molecules, causing dissolution [32].

The increasing porosity corresponded with an increase in roughness and surface nano-topography (figure 5). The pores formed from treatments 1 and 3 were distinctively round and the pore walls were made of round fibrils. In particular, for reactions times greater than or equal to 30 s in treatments 1 and 3, the pore wall thickness, as quantified by the diameter of the fibrils surrounding the pores, steadily decreased to an average of 110 nm at 90 s (table 1) while the average pore size remained comparable at 0.51 ± 0.17 µm and 0.42 ± 0.04 µm, respectively (figure 5*c*1–*e*1 and 5*c*3–*e*3). We note here that the brief treatment duration under ambient temperature means that these treatments only modified the surface of the samples as shown in electronic supplementary material, figure S3. The modified surface can be partially polished by tissue paper as shown in treatment 2 (electronic supplementary material, figure S3*c*1).

**Table 1.** Diameter of fibrils surrounding the pores on treated PEEK surfaces.

reaction time (s)	diameter of fibrils on porous surface (pore wall thickness, *μ*m)		
	treatment 1	treatment 2	treatment 3
10	0.20 ± 0.04	0.29 ± 0.11	0.19 ± 0.08
30	0.24 ± 0.08	0.27 ± 0.10	0.26 ± 0.10
60	0.13 ± 0.04	0.21 ± 0.09	0.12 ± 0.04
90	0.11 ± 0.03	0.25 ± 0.11	0.11 ± 0.02

Both surface nano-topography and the introduction of charged sulfonate groups can affect hydrophilicity. To distinguish the morphological effect versus the chemical compositional effect of sulfonation on the subsequent hydrophilicity analysis (discussed in §3.3), treatment 2 incorporated a final polishing step to treatment 1 to gently homogenize the surface topography without removing the polar SPEEK layer.

Indeed, for reaction durations long enough to generate nano-topography (greater than or equal to 30 s), the surface morphology of the polished samples from treatment 2 was found to be highly comparable, with less distinct pores surrounded by flattened fibrils of similar average diameters (table 1 and figure 5*c*2–*e*2). The surface morphology was found to have a strong impact on the water contact angle results, which will be described in §3.3.

Young's modulus of untreated PEEK was 3.2 ± 0.1 GPa, which is consistent with the literature value [9]. Young's moduli of all treated PEEK samples were comparable to those of the untreated control (figure 6*b* and table 2), suggesting that the treatments and surface porosity generated by the treatments do not affect the mechanical properties of the treated PEEK samples.

**Table 2.** Young's moduli of treated PEEK samples compared with the untreated control and the literature [9].

samples	Young's modulus, *E* (GPa)
untreated PEEK	3.2 ± 0.1
T1 (30 s)	3.2 ± 0.1
T1 (90 s)	3.2 ± 0.1
T2 (30 s)	3.3 ± 0.1
T2 (90 s)	3.3 ± 0.1
T3 (30 s)	3.2 ± 0.1
T3 (90 s)	3.1 ± 0.1

### 3.3. Effect of sulfonation on surface hydrophilicity

**Figure 7.**
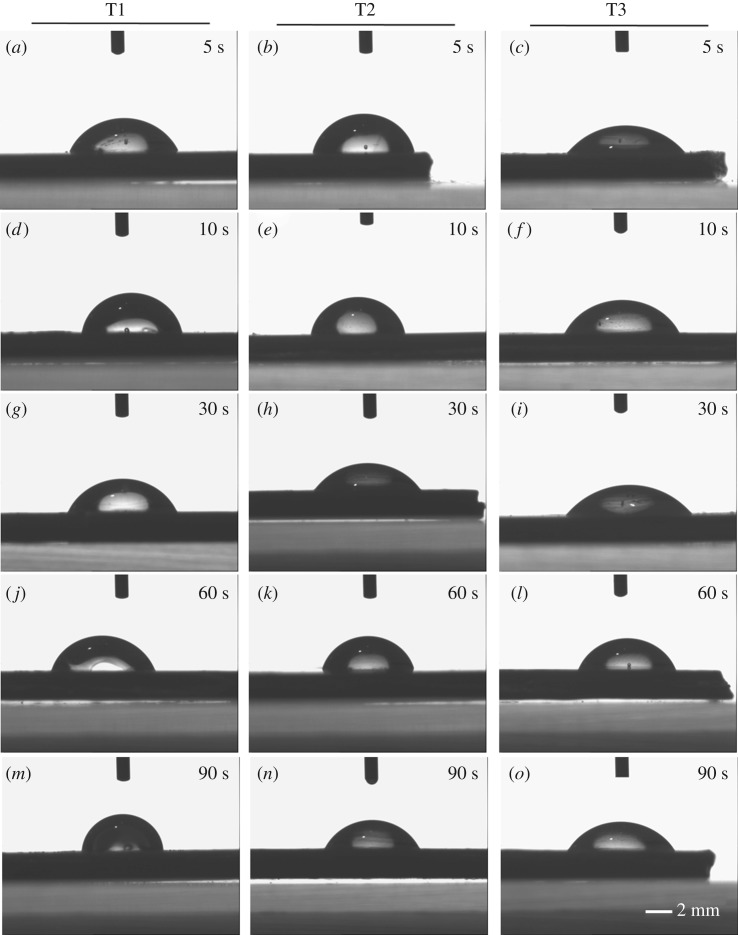
Water contact angle on PEEK surfaces modified by different sulfonation procedures and reaction durations. (*a*, *d*, *g*, *j*, *m*) Treatment 1. (*b*, *e*, *h*, *k*, *n*) Treatment 2. (*c*, *f*, *i*, *l*, *o*) Treatment 3. (*a–c*): 5 s, (*d–f*): 10 s, (*g–i*): 30 s, (*j–l*): 60 s, (*m–o*): 90 s. Scale bar: 2 mm.

**Figure 8.**
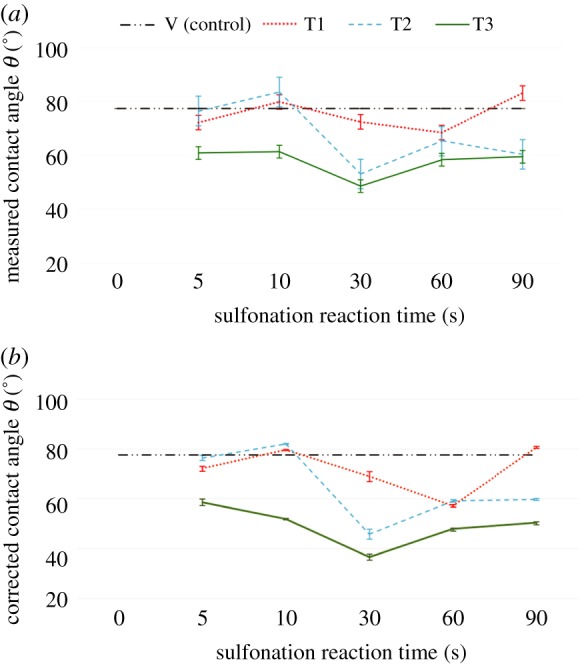
(*a*) Measured and (*b*) corrected water contact angle on PEEK surfaces modified by different sulfonation treatments and reaction durations. Corrected water contact angle values are calculated using the Wenzel equation, taking into account surface roughness due to porosity generated by the treatments. T1: treatment 1. T2: treatment 2. T3: treatment 3. *V* (control): contact angle on untreated virgin PEEK surface at 77.6 ± 0.3°. (Online version in colour.)

The hydrophilicity of the treated surfaces was analysed using water contact angle (*θ*) (figures 7 and 8). Contact angle is a key parameter to study the surface properties of materials. Changes in the contact angle of a polymer material indicate changes in its surface chemistry [33]. PEEK has relatively low hydrophilicity; the contact angle of untreated PEEK in the literature is between 70° and 90° [17,33]. In this work, the water contact angle of untreated virgin PEEK samples was 77.6 ± 0.3°.

Increasing the reaction time from 5 s to 90 s increases the number of polar sulfonate groups introduced on the PEEK surface. The increasing polarity was expected to have a proportional effect on hydrophilicity as expressed by a decreasing trend in the water contact angle on the treated PEEK [14]. However, sulfonation also created a significantly roughened, nano-porous PEEK surface (figure 5). A water droplet on a rough surface with nanometre features can either penetrate the porous grooves or suspend above the grooves (in the case of superhydrophobicity), thereby affecting the measured contact angle values [34]. Moreover, longer sulfonation reaction times of 60–90 s generated a significantly rougher, more porous surface than the shorter reaction times (figures 5 and 6 and table 1), and this is expected to compromise the effect of longer sulfonation time on wettability. Hence, determining an optimal reaction time for improving PEEK hydrophilicity while creating surface nano-topography for optimal cell–implant interaction would be desirable.

To account for the roughened porous surface of the samples in assessing the water contact angle values, we adopted the Wenzel equation (cosθ* *=* *R* cos*θ*) to correct the measured value against the roughened wetted surface under the Wenzel wetting state, where *R* is the ratio of the roughened wet surface area to its projection on the apparent solid plane (see Material and methods and electronic supplementary material, figure S2) [26]. The measured water contact angles of the treated samples are presented in figure 8*a*, and the correspondingly corrected water contact angle graphs are presented in figure 8*b*. The Wenzel equation has a practical relevance: the sign of the cosine function predicts that hydrophilic surfaces with contact angles below 90° become more hydrophilic by roughening; therefore, the corrected water contact angle values are lower than the measured contact angle values [28]. Discussions given below on contact angle results refer to the corrected water contact angle values based on the Wenzel equation.

Treatment 1 (T1) uses concentrated sulfuric acid alone and was found to produce little change in the water contact angle of the PEEK surface for all of the reaction times when compared with the untreated virgin PEEK (table 3 and figure 8*b*, T1 and V). The water contact angle was found to be between 57.2 ± 1.6° and 80.6 ± 2.8°, in comparison with the untreated sample at 77.6 ± 0.3°; the changes in hydrophilicity were statistically insignificant (*p*-value > 0.1) for all T1 reaction times. This is in agreement with previous findings by Zhao *et al*. [24], in which a microwave-assisted, short reaction time (minutes) sulfonation procedure was used with concentrated sulfuric acid alone. They similarly observed no improvement in surface hydrophilicity. Nevertheless, significantly improved osteoconduction from the modified porous PEEK surface was observed [24]. In addition, treatment 2 (T2) also produced insignificant changes in water contact angle in comparison with that of the untreated PEEK (figure 8, T2 and V, *p*-value > 0.05). The results were comparable to those from T1 over the same reaction time of less than or equal to 10 s. Taken together, these results show that ambient-temperature sulfonation treatments T1 and T2 were insufficient to affect PEEK hydrophilicity.

**Table 3.** Water contact angle on PEEK surfaces treated by different sulfonation procedures and reaction times.

reaction time (s)	water contact angle (°) based on Wenzel equation		
	treatment 1	treatment 2	treatment 3
5	72.1 ± 1.6	76.4 ± 1.0	58.6 ± 1.3
10	79.6 ± 1.9	82.0 ± 0.2	51.9 ± 0.3
30	68.9 ± 1.0	45.9 ± 2.0	36.7 ± 1.2
60	57.2 ± 1.6	59.2 ± 0.5	47.8 ± 0.6
90	80.6 ± 2.8	59.8 ± 0.4	50.2 ± 0.6

Treatment 3 (T3) was found to be the most effective at reducing water contact angle *θ* (*p*-value = 0.00067). At the shortest sulfonation time of 5 s, the contact angle decreased to 58.6 ± 1.3°, a 25% reduction in comparison with the untreated control (figures 7 and 8). T3 generated the lowest *θ* value of all treatments; this was found to be 36.7 ± 1.2° at 30 s reaction time, a 53% improvement in hydrophilicity from that of untreated PEEK (figure 8, T3 and V; table 3).

In comparison with T1 and T2, T3 further exposes the acid SPEEK-H to 20 s immersion in 6 wt% NaOH and converted the SPEEK-H to its sodium salt, SPEEK-Na (figure 2). NaOH is often used after PEEK sulfonation in fuel cell applications to demonstrate the ion exchange capacity of the resultant SPEEK-H film. The effectiveness of SPEEK-Na in improving PEEK hydrophilicity as observed here is in good agreement with Zhao *et al*. [24], in which SPEEK-Na was produced by NaOH etching, and the resultant water contact angle was also found to decrease from 78 ± 9° to 43 ± 3°.

For bone implant applications, converting SPEEK-H to SPEEK-Na has several added advantages. The salt form SPEEK-Na has been found to exhibit better thermal stability than the acid form SPEEK-H [32]. Thermal stability may not be directly relevant for *in vivo* environments under physiological conditions, though it is a potentially useful feature during processing and modifications of the material prior to implantation. Furthermore, the additional 20 s step using NaOH can quickly neutralize any residual acid from sulfonation. Residual sulfuric acid from sulfonation of PEEK has been found to inhibit the growth of osteoblasts and give rise to slower bone formation [35]. T1 and T2 had a respective final pH of 6.31 ± 0.06 and 6.38 ± 0.08, whereas the pH at the end of T3 was close to neutral, at 6.92 ± 0.04.

We note that the surface hydrophilicity did not improve with the increasing surface porosity and nano-topography as the reaction time increased. The most hydrophilic SPEEK surface was found to be at 30 s of sulfonation for both T2 and T3 (figure 8 and table 3). At 30 s treatment duration, T3 generated pores with smooth and broad surrounding surfaces (figure 5, c3), whereas the treated surfaces at longer reaction durations of 90 s generated heterogeneous roughness on the surface at the micro- and nano-scale (figure 5*e*1,*e*3). The heterogeneities on the treated surfaces at longer reaction times could have contributed to the possibility that some regions of the surface exhibit full water penetration into the topography (the Wenzel wetting state), while other regions may exhibit partial or no penetration (the Cassie–Baxter wetting state) [28], thereby increasing the complexity of the surface wetting analysis, which should be further investigated in future work. Here, a water drop on the roughened surface may interact with a heterogeneous surface composed of the solid material and air trapped in the micro/nano-topography, thereby increasing the contact angle on the porous heterogeneous samples generated at reaction times of 60–90 s (porosity ≥ 65%, figure 6). Hence, the optimal sulfonation reaction time to generate a nano-porous PEEK surface with the highest hydrophilicity was determined as 30 s in this work. Future work will expand the contact angle analysis using the Lifshitz–van der Waals/acid–base (LW-AB) approach to assess the surface free energy of the treated PEEK samples to further understand the thermodynamics of the biomaterial in relevance for biological systems.

Both hydrophilicity and nano-topography are highly favourable features of a bone implant interface, but there is no current consensus on their relative importance for bone anchorage [36,37]. A recent study indicated that increased implant nano-topography may be more important for osseointegration than hydrophilicity, but quantification in real-life surfaces is highly complex and the relative roles of nano- and macroscopic geometrical cues are still under debate. For example, a recent study has demonstrated that nano-scale contact guidance could be overruled by meso-scale substrate curvature and geometrical cues of up to 10× cell size can play a dominant role in directing bone marrow stroma cell migration [38]. Hence, further research is required to comprehensively determine the relative biomaterial interface effects in future cell culture studies [28,37].

Most importantly, the short reaction time and ambient-temperature sulfonation treatment 3 developed in this work is significantly more effective than many conventional high-energy modification techniques reported to date. Treatment 3 at 30 s reduced the PEEK water contact angle from 77.6 ± 0.3° to 36.7 ± 1.2°. This is better than conventional physical treatments, such as gas plasma etching (e.g. oxygen and ammonia), or electron beam deposition of bioactive substrates, such as titanium . For example, using oxygen plasma etching, Poulsson *et al*. [39] reduced the water contact angle on extrusion-machined PEEK from 85.47 ± 7.90° to 60.28 ± 8.09°, and Han *et al*. [40] employed electron-beam deposition of titanium on PEEK and reduced the water contact angle from 71 ± 5.1° to 54 ± 2.4°.

## 4. Conclusion

This work focuses on developing a new, fast and economical treatment methodology to increase the hydrophilicity of PEEK and systematically investigates a series of rapid, ambient-temperature sulfonation procedures. The treatment presented in this work has demonstrated major applicability for introducing surface roughness and hydrophilicity. Three treatments were tested with varying reaction times of 5–90 s: (T1) concentrated H_2_SO_4_; (T2) concentrated H_2_SO_4_ followed by water rinsing and polishing; (T3) concentrated H_2_SO_4_ followed by water rinsing, 20 s immersion in NaOH and water rinsing again. T3 was the most effective, in which the 30 s sulfonation reaction time was found to be more beneficial than those of conventional treatments, reducing the PEEK water contact angle from 77.6 ± 0.3° to 36.7 ± 1.2°. Furthermore, surface porosity and nano-topography increased with increasing sulfonation reaction time and adversely affected hydrophilicity. Therefore, the optimal sulfonation reaction time was determined at 30 s to generate a nano-porous PEEK surface with the highest hydrophilicity. Future work will investigate the cell–material interaction and cytotoxicity of the sulfonated PEEK materials to pave the way for improved PEEK implant applications.

## Supplementary Material

Figure S1

## Supplementary Material

Figure S2

## Supplementary Material

Figure S3

## Data accessibility

All data relating to this paper are contained in the paper and/or electronic supplementary material.
