# Supplementary figures and images for: PEEK surface modification by fast ambient-temperature sulfonation for bone implant applications
Source: J R Soc Interface. 2019 Mar 6;16(152):20180955. doi: 10.1098/rsif.2018.0955 (PMC6451405; doi:10.1098/rsif.2018.0955)

(a)

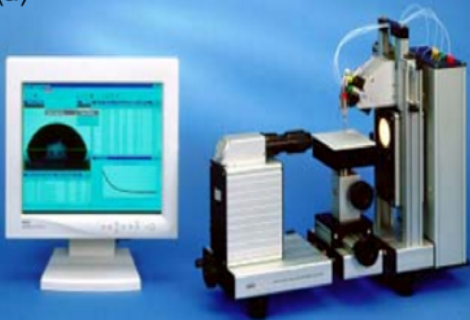

(b) Untreated PEEK

$$\theta = 77.6 \pm 0.3^\circ$$

— 2 mm

Supplement: Figure S1 [file rsif20180955supp1.pdf]

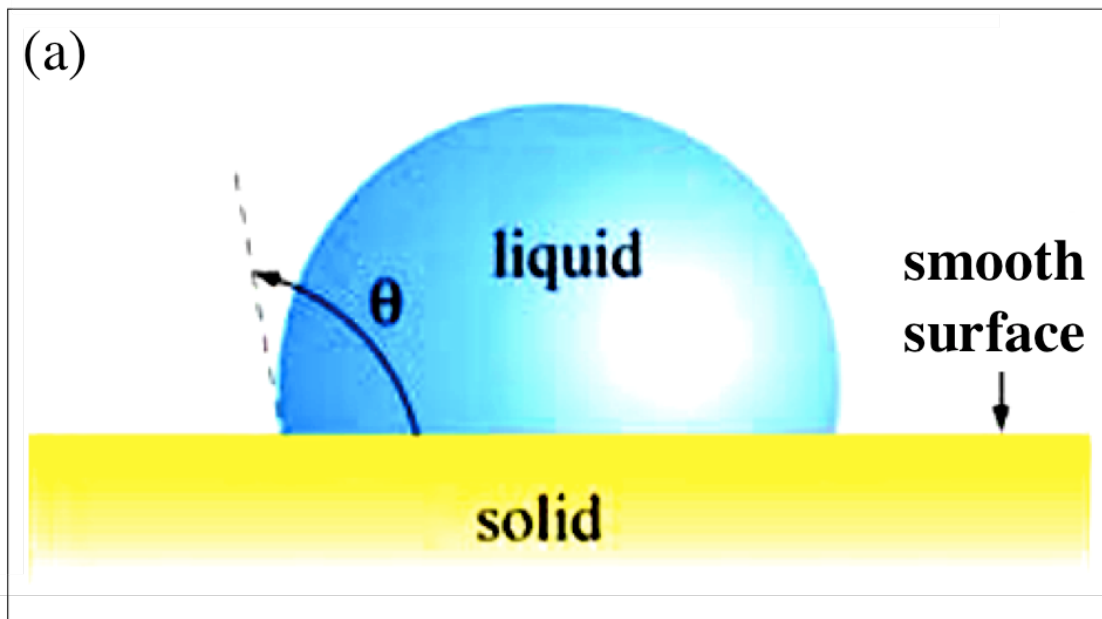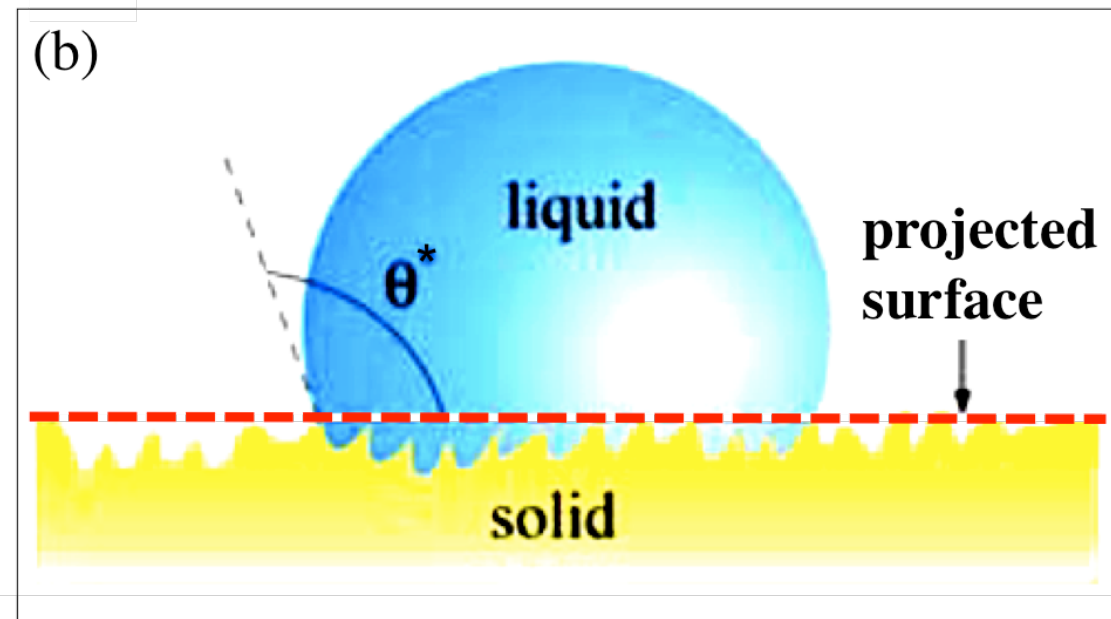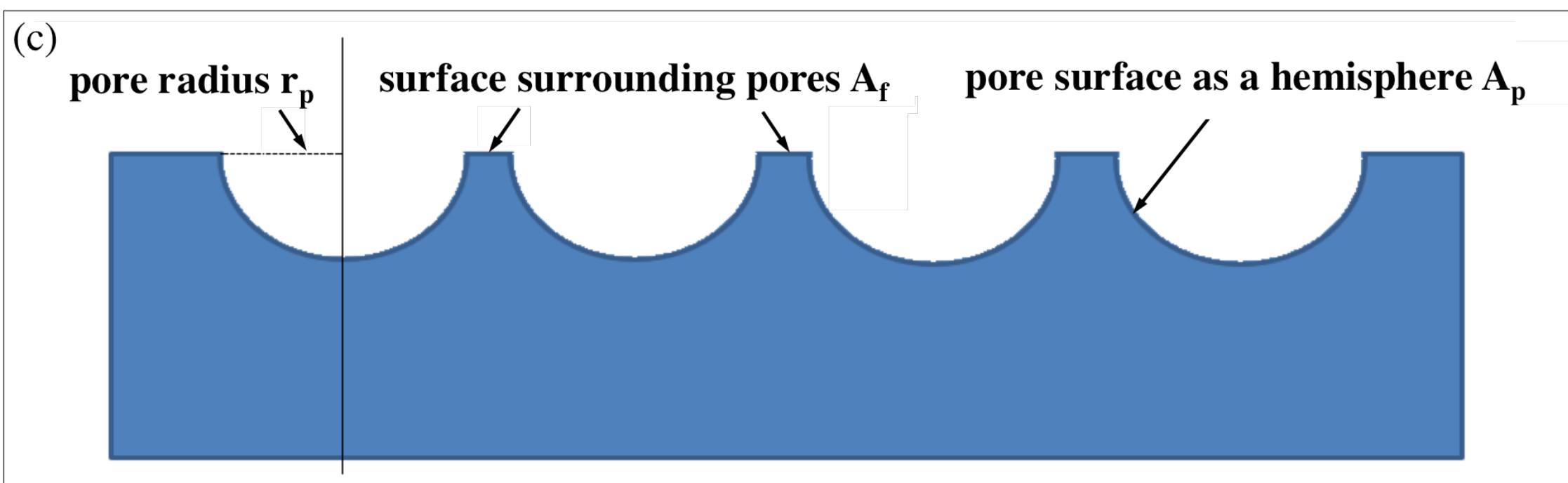

Supplement: Figure S2 [file rsif20180955supp2.pdf]

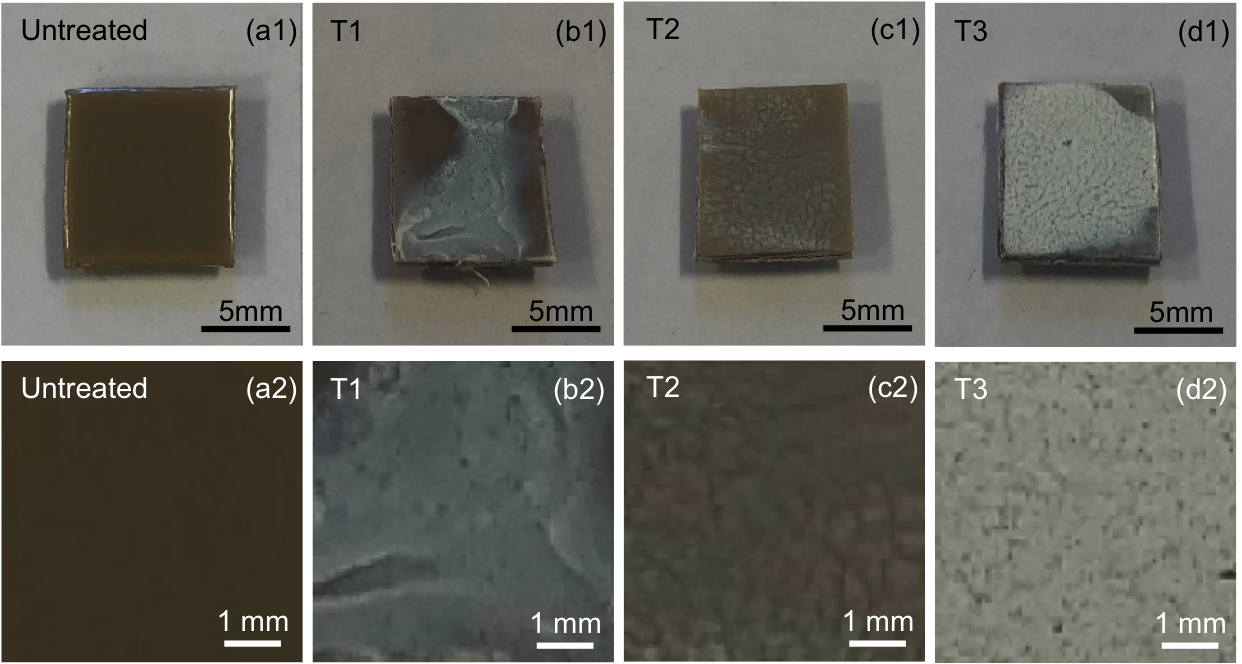

Supplement: Figure S3 [file rsif20180955supp3.png]
